# State-dependent value representation: evidence from the striatum

**DOI:** 10.3389/fnins.2014.00193

**Published:** 2014-07-15

**Authors:** Christopher J. Burke, Jean-Claude Dreher, Ben Seymour, Philippe N. Tobler

**Affiliations:** ^1^Laboratory for Social and Neural Systems Research, Department of Economics, University of ZurichZurich, Switzerland; ^2^Neuroeconomics Laboratory: Reward and Decision-Making, CNRS, UMR 5229, Université de Lyon, Université Claude Bernard Lyon 1Lyon, France; ^3^Center for Information and Neural Networks, National Institute for Information and Communications TechnologyOsaka, Japan; ^4^Computational and Biological Learning Lab, Department of Engineering, University of CambridgeCambridge, UK

**Keywords:** conditioned taste aversion, stress, psychological, dopamine, addiction, reward, translation

The ability to distinguish good from bad options, to approach the former and avoid the latter, forms the basis of successful behavior. This ability is expressed in value-based decisions, which in turn is thought to depend largely on the process of reinforcement learning. In order to adaptively determine the value of different actions, organisms need to take external as well as internal states into account (e.g., Rangel et al., 2008). For example, finding shelter may be more valuable in a cold environment than in a warm environment. Internal states can also affect valuation, as illustrated for instance by salt appetite (Berridge et al., 1984; Tindell et al., 2009; Robinson and Berridge, 2013): In the normal (non-salt-deficient) state, rats do not usually ingest extremely salty solutions or approach cues that predict them. However, in a salt-deficient state, they do. This pattern of behavior is compatible with the notion that state information can have such a profound impact on value computation that a previously bad option becomes good (Dayan and Berridge, 2014).

Since its inception in the seventeenth century, economic choice theory has gradually come to recognize the importance of internal and external states on valuation. While it was initially thought that a given monetary unit was worth the same no matter how wealthy one is (Pascal), it was later proposed that the value of a given monetary unit is greater when one is in a state of poverty as compared to one of affluence (Bernoulli). In the last century, researchers found that our expectations also affect valuation and accounted for this finding by incorporating reference points into value functions (e.g., prospect theory: Kahneman and Tversky, 1979; see also Koszegi and Rabin, 2006). For example, we value a salary raise of USD 200 less when we originally expected to receive a raise of USD 400 than when we did not expect to receive any raise at all. Thus, the value of an option can also depend on cognitive states.

The states and other variables that influence valuation are manifold and include not only financial status and expectations, but also mood, emotion, motivation, previous learning, and social aspects. Still, little is known about how state information influences valuation at the neural level. Two recent publications (McCutcheon et al., 2012; Porcelli et al., 2012) addressed this question. The two studies used different techniques (dopamine voltammetry vs. functional magnetic resonance imaging), different model organisms (rats vs. humans), and different value-impacting state parameters (previous learning vs. stress). Despite these differences, both studies found that striatal value signals are state-dependent.

In the first study, McCutcheon et al. (2012) measured dopamine release in the nucleus accumbens shell following intra-oral infusion of sucrose. In half of the rats, sucrose was rendered aversive by pairing it with induced nausea (via injection of lithium chloride just after sucrose consumption). In the other half of the rats, nausea induction and sucrose consumption occurred on different days, so sucrose remained appetitive. As expected, aversive sucrose elicited fewer appetitive and more negative orofacial responses. Neurobiologically, unlike appetitive sucrose, aversive sucrose reduced accumbens dopamine concentration compared to baseline, even though the sensory properties of the sucrose were held constant in the two conditions. This finding converges with several previous reports of reduced dopamine firing and concentrations induced by aversive stimuli (for review, see McCutcheon et al., 2012). Conversely, appetitive sucrose elicited a (weak) increase in dopamine, in line with previous voltammetry data (Roitman et al., 2008) and a wealth of previous findings implicating dopamine in reward processing (for a review, see Daw and Tobler, 2013).

The reduction in accumbens dopamine concentration in response to aversive sucrose shows that learning can change the value of a primary appetitive stimulus and provides a pharmacological foundation to the reduction in striatal activation observed in imaging studies in which participants' reward expectations were disappointed (e.g., McClure et al., 2003; O'Doherty et al., 2003; Pessiglione et al., 2006; Burke et al., 2010; Kahnt et al., 2012).

In the second study, Porcelli and colleagues investigated the flexibility of reward processing in response to induced stress (an internal state factor). They adapted the cold pressor task, an established stress induction procedure (Schwabe et al., 2008), for use in the scanner. They used MRI-compatible gelpacs to subject half of the participants to stress-inducing cold temperatures and half to room temperature (for details on the procedure, see Porcelli, 2014). Both groups then performed a card-guessing task in which they could win $5 or $0.50, or lose $0.25 or $2.50. The asymmetry between the gain and loss domains aimed to ensure that the impact of reward and punishment on behavior and brain activity was similar and thus to compensate for the fact that people generally exhibit loss aversion (another feature of prospect theory; Kahneman and Tversky, 1979). Throughout the experiment, the level of salivary cortisol (a stress-inducing hormone) was measured at 15 minute intervals using an oral swab.

The adapted cold pressor task resulted in greater feelings of discomfort and a higher cortisol level in the stress induction group than in the control group. However, the most striking results from this study pertain to the striatal BOLD response: Responses to rewards as compared to punishments were much larger in the caudate and putamen in the control group than in the stress induction group. Participants in the latter group only showed reward-related activation in this region when outcomes were of high magnitude, suggesting that stress causes a desensitization of the reward network. In addition, the reduction in response to rewards under stress was observed in the dorsal, but not the ventral striatum. These regions have been shown to correlate with reward learning in a manner consistent with computational models of reinforcement learning (specifically, the actor-critic model proposed by Barto, 1995), with dorsal actor regions processing action contingencies that guide future choices, and ventral critic regions making predictions about future rewards (O'Doherty et al., 2004). If stress causes a desensitization to reward in the “actor” areas of the striatum, action-outcome contingencies may not be processed accurately enough to guide future choices, forcing the organism to rely on more habitual responses. This may manifest itself in a return to otherwise suboptimal reward-seeking behavior, such as the relapses commonly seen in recovering addicts (Everitt and Robbins, 2005).

Moreover, one could hypothesize that stress, which by itself increases tonic levels of dopamine (e.g., Inoue et al., 1994), may prevent the detection of phasic reductions in dopamine that could be elicited by stimuli that predict negative drug-related effects (see also Weiss et al., 2001; Schultz, 2011). This notion would predict that stress reduces sensitivity to losses through a dopamine-dependent mechanism. Incidentally, one possible target region for implementing this mechanism is suggested by the Porcelli study (Porcelli et al., 2012), which reports reduced magnitude discrimination under stress in the inferior frontal gyrus. Given that the inferior frontal gyrus appears to play a role in response inhibition and cognitive control (e.g., Bari and Robbins, 2013), it might be worth investigating the role of dopamine on punishment sensitivity under withdrawal-induced stress in that region.

Taken together, the two papers discussed here support the notion that reward processing in the brain is flexible and highly dependent on internal and external states. The interaction between the environment and internal states allows organisms to prioritize their goals and adapt their behavior accordingly. We suggest that this inherent flexibility can be detrimental when reward and learning systems are artificially challenged, for example, through the use of addictive drugs. While the use of drugs themselves may affect internal states (such as by tonically enhancing dopamine levels), subsequent withdrawal may induce stress, causing a change in behavior, e.g., in the form of a shift toward habitual responding and enhanced drug-seeking. Moreover, state processes could interact with drug effects and further research may wish to investigate how this interaction contributes to addiction and relapse. While the two papers (McCutcheon et al., 2012; Porcelli et al., 2012) give some leads for that endeavor they more generally highlight the importance of external and internal states for brain and behavior.

## Conflict of interest statement

The authors declare that the research was conducted in the absence of any commercial or financial relationships that could be construed as a potential conflict of interest.
